# Mutation analysis of the *NSD1 *gene in patients with autism spectrum disorders and macrocephaly

**DOI:** 10.1186/1471-2350-8-68

**Published:** 2007-11-14

**Authors:** Joseph D Buxbaum, Guiqing Cai, Gudrun Nygren, Pauline Chaste, Richard Delorme, Juliet Goldsmith, Maria Råstam, Jeremy M Silverman, Eric Hollander, Christopher Gillberg, Marion Leboyer, Catalina Betancur

**Affiliations:** 1 Laboratory of Molecular Neuropsychiatry, Mount Sinai School of Medicine, New York, USA.; 2 Department of Psychiatry, Mount Sinai School of Medicine, New York, USA.; 3 Seaver Autism Research Center, Mount Sinai School of Medicine, New York, USA.; 4 Department of Child and Adolescent Psychiatry, Goteborg University, Goteborg, Sweden.; 5 AP-HP, Hôpital Robert Debré, Service de Psychopathologie de l'Enfant et de l'Adolescent, Paris, France.; 6 INSERM U841, Institut Mondor de Recherche Biomedicale, Psychiatric Genetics, Créteil, France.; 7 Université Paris 12, Faculté de Médecine, Créteil, France.; 8 Institute of Child Health, London, UK.; 9 AP-HP, Groupe Hospitalier Henri Mondor – Albert Chenevier, Department of Psychiatry, Créteil, France.; 10 INSERM U513, Créteil, France.

## Abstract

**Background:**

Sotos syndrome is an overgrowth syndrome characterized by macrocephaly, advanced bone age, characteristic facial features, and learning disabilities, caused by mutations or deletions of the *NSD1 *gene, located at 5q35. Sotos syndrome has been described in a number of patients with autism spectrum disorders, suggesting that *NSD1 *could be involved in other cases of autism and macrocephaly.

**Methods:**

We screened the *NSD1 *gene for mutations and deletions in 88 patients with autism spectrum disorders and macrocephaly (head circumference 2 standard deviations or more above the mean). Mutation analysis was performed by direct sequencing of all exons and flanking regions. Dosage analysis of *NSD1 *was carried out using multiplex ligation-dependent probe amplification.

**Results:**

We identified three missense variants (R604L, S822C and E1499G) in one patient each, but none is within a functional domain. In addition, segregation analysis showed that all variants were inherited from healthy parents and in two cases were also present in unaffected siblings, indicating that they are probably nonpathogenic. No partial or whole gene deletions/duplications were observed.

**Conclusion:**

Our findings suggest that Sotos syndrome is a rare cause of autism spectrum disorders and that screening for *NSD1 *mutations and deletions in patients with autism and macrocephaly is not warranted in the absence of other features of Sotos syndrome.

## Background

Sotos syndrome (MIM 117550) is a childhood overgrowth syndrome characterized by distinctive facial features including prominent forehead, down-slanted palpebral fissures and pointed chin, macrocephaly and learning disabilities. Sotos syndrome is caused by haploinsufficiency of the *NSD1 *(nuclear receptor binding SET domain protein 1) gene [1], which encodes a coregulator of nuclear receptors that can activate or repress transcription [2]. Point mutations are identified in the majority of non-Japanese patients with Sotos syndrome (~80%), whereas 5q35 microdeletions encompassing *NSD1 *are the major cause among Japanese patients (>50%) [3,4]. Most cases of Sotos syndrome are sporadic, but occasional familial cases have been reported, with dominant inheritance. Affected children usually exhibit developmental delay, and speech delay is common. In addition, autism spectrum disorders or autistic features have been described in a number of patients with Sotos syndrome [5-13].

Autism is a behaviorally defined neurodevelopmental syndrome characterized by social and communication deficits, and the presence of restricted and repetitive behaviors and interests, with onset in the first three years of life. Recent epidemiological studies indicate that autism is a common disorder, affecting as many as 2 in 1000 children [14]. The prevalence of all autism spectrum disorders, which include autism, pervasive developmental disorder not otherwise specified (PDD-NOS) and Asperger syndrome, is estimated at 6 per 1000 [14]. Autism is approximately four times more frequent in males than in females. Family and twin studies have shown that genetic factors play a major role in the susceptibility to autism [15] but genetic heterogeneity has made the identification of the genes involved difficult [16]. Monogenic disorders such as fragile X syndrome and other forms of X-linked mental retardation, tuberous sclerosis complex, neurofibromatosis, and various rare metabolic disorders are identified in a small percentage of patients [17]. Cytogenetically visible chromosomal aberrations are identified in approximately 5% of affected individuals [10], while recent higher-resolution whole-genome analyses using array-based technologies have revealed genomic imbalances in at least 10% of cases [18,19]. However, the underlying cause remains unknown in the majority of patients.

Autism is associated with macrocephaly in approximately 20% of cases [20,21]. Although macrocephaly is one of the most widely replicated neurobiological findings in autism, its pathogenesis remains unknown. The description of several cases of Sotos syndrome in patients with autism and macrocephaly [5-13] suggests that *NSD1 *could be involved in other cases of autism. Thus, the aim of this study was to assess the frequency of *NSD1 *mutations in cases of autism spectrum disorder associated with macrocephaly, defined as an occipitofrontal head circumference (HC) 2 SD or more above the mean. We screened *NSD1 *by direct sequencing in 88 subjects with autism spectrum disorder and macrocephaly. In addition, we searched for deletions of the *NSD1 *gene using multiplex ligation-dependent probe amplification (MLPA). Our results showed no point mutations or deletions of *NSD1*, indicating that Sotos syndrome is a rare cause of autism spectrum disorders with macrocephaly.

## Methods

### Patients

A total of 88 patients with an autism spectrum disorder and macrocephaly (HC ≥+2 SD) were included in the study. Among these, 49 were recruited by the Paris Autism Research International Sibpair (PARIS) study at specialized clinical centers in France, Sweden, Norway, Italy, Belgium, Austria, and the United States, and 39 were collected at the Mount Sinai School of Medicine and/or the Autism Genetic Resource Exchange (AGRE) [22]. Patients were identified from a larger pool of 462 families for which head circumference measures were available. All patients with a HC ≥+2 SD were included in the study; in families with two or more siblings with an autism spectrum disorder and macrocephaly, one individual was chosen at random for the mutation screening.

The patients from the PARIS study included 45 males and 4 females from 16 multiplex families (with two or more affected siblings) and 33 simplex families (sporadic cases), with a mean age at the last evaluation of 10.5 ± 5.8 years (range 3.5–26). All patients were evaluated by experienced psychiatrists or child neurologists; diagnoses were based on clinical evaluation and DSM-IV and ICD-10 criteria. In addition, patients with autistic disorder were assessed with the Autism Diagnostic Interview-Revised (ADI-R) [23] and those with Asperger syndrome were assessed with the Asperger Syndrome Diagnostic Interview (ASDI) [24]. Forty-five individuals met criteria for autistic disorder, 2 for PDD-NOS and 2 for Asperger syndrome. There were 35 patients with mental retardation and 22 with limited or no language. Laboratory tests to rule-out medical causes of autism included standard karyotyping, fragile X testing, and metabolic screening; brain imaging and EEG were performed when possible. Patients diagnosed with medical disorders such as fragile X syndrome or chromosomal abnormalities were excluded from the study. There were 41 individuals of Caucasian origin, 2 Black, 1 Asian and 5 of mixed ethnicity. The study was approved by the research ethics boards of the collaborating institutions. Informed consent was obtained from all families participating in the study.

Thirty-nine patients were recruited by the Seaver Autism Research Center (SARC) at Mount Sinai, New York, co-recruited by the SARC and AGRE, or obtained from AGRE. The patients included 31 males and 8 females from 35 multiplex families and 4 simplex families, with a mean age at the last evaluation of 10.5 ± 4.2 years (range 5–24). The ADI-R was used to assess affected subjects. There were 35 individuals with autism, 1 with borderline autism, 2 with Asperger syndrome and 1 with PDD-NOS. The term 'borderline autism' (or 'not quite autism') refers to individuals who are no more than one point away from meeting autism criteria according to the ADI-R on any or all of the 3 main domains of autistic impairment – social, communication, and repetitive behavior – and meet the age of onset criterion (before 36 months); or individuals who meet criteria on all 3 domains but do not meet the age of onset criterion. Seventeen patients had mental retardation and 16 had limited or no language. Subjects with co-morbid genetic disorders (fragile X syndrome, tuberous sclerosis, or chromosomal anomalies) were excluded. There were 32 individuals of Caucasian origin, 1 Black, 1 Asian, and 5 of mixed or unknown ethnicity. All parents provided written informed consent. The study was approved by the local institutional review board.

HC among the 88 subjects included in the study ranged from +2 to +9.6 SD for age and sex. There were 68 subjects with a HC between +2 and +2.9 SD, 18 with a HC between +3 and +4 SD, and only 2 subjects with a HC greater than +4 SD. The patient with the largest HC (+9.6 SD) among all the study subjects was later found to have a *PTEN *mutation [25].

### *NSD1 *mutation analyses

The *NSD1 *gene consists of 23 exons, the first of which is non coding. All exons (including exon 1), intron-exon boundaries and flanking intronic sequences were analyzed for mutations by direct sequencing of forward and reverse strands. When variants not described previously were detected, the DNA of parents and unaffected siblings (when available) was analyzed by direct sequencing for the specific base change identified in the proband.

### *NSD1 *microdeletion analyses

In order to identify microdeletions of the *NSD1 *gene we performed MLPA [26] using a commercially available kit, according to the manufacturer's protocol (SALSA P026B Sotos; MRC-Holland, Amsterdam, The Netherlands). The P026B probe set contains 24 *NSD1 *probes, including one probe in the promoter region (1 Kb 5' of exon 1) and one probe for each of the 23 exons. The kit also contains six probes for five neighboring genes on 5q31-35 (*IL4*, *IL12B*, *FGFR4*, *FLT4 *and *TRIM52*), as well as 12 control probes in other chromosomes. Electrophoresis of PCR products was performed on an ABI 3730 sequencing platform (Applied Biosystems, Foster City, CA, USA) and the resultant traces were analyzed using the software GeneMarker (SoftGenetics, State College, PA, USA).

## Results

### *NSD1 *mutation analyses

Genomic DNA from 88 unrelated individuals with autism spectrum disorders and macrocephaly was screened for intragenic *NSD1 *mutations by sequencing analysis. No frameshift or nonsense mutations were identified in any sample. Three missense variants, R604L, S822C and E1499G, were each identified in one individual (Table 1). R604L, in exon 5, was described previously in one patient referred for genetic testing of Sotos syndrome, and was classified as a variant of unknown significance [27]. S822C, also in exon 5, and E1499G in exon 10, have not been described previously. None of these variants is in a known functional domain of the protein [28]. Segregation analysis showed that all three variants were inherited from reportedly healthy parents and in two cases were present also in unaffected siblings, indicating that they are likely nonpathogenic. Furthermore, we identified two synonymous variants and two intronic substitutions present in one patient each, which were also inherited from unaffected parents. Table 1 shows all the sequence changes identified in our patients, including several new polymorphisms identified in this study.

**Table 1:** Sequence variants identified in the *NSD1 *gene in 88 patients with autism spectrum disorders and macrocephaly

Location and Nucleotide Change	Protein Change	Frequency	Inheritance	Previously described^1^
Intron 1				
IVS1+6T→C	...	1 T/C	Paternal	No
Exon 2				
c.339C→T	C113C	1 T/T	Paternal/maternal, present in 1 sib with autism and 1 unaffected sib	[27, 33]
Exon 5				
c.1482C→T	C494C	38 C/T, 2 T/T		rs1363405
c.1811G→T	R604L	1 G/T	Paternal (no siblings)	[27]
c.1749G→A	E583E	19 G/A, 1 A/A		rs3733874
c.1792T→C	L598L	3 T/C		rs28932176
c.840G→T	V614L	19 G/T, 1 T/T		rs3733875
c.2071G→A	A691T	5 G/A		rs28932177
c.2176T→C	S726P	22 T/C, 2 C/C		rs28932178
c.2465C→G	S822C	1 C/G	Maternal, present in 1 unaffected sib	No
c.2835T→C	S945S	1 T/C	Maternal (absent in 1 sib with autism)	No
c.3106G→C	A1036P	5 G/C		rs28932179
c.3705T→C	N1235N	13 T/C		rs28932181
Exon 10				
c.4496A→G	E1499G	1 A/G	Paternal, present in 1 unaffected sib	No
Intron 14				
IVS14-45C→G	...	1 C/G	Maternal, present in 1 unaffected sib	No
Intron 17				
IVS17-22G→A	...	16 G/A		[51, 52]
Exon 23				
c.6750G→A	M2250I	16 G/A		rs35848863
c.6782T→C	M2261T	16 T/C		rs34165241
c.6829C→T	L2277L	19 C/T, 1 T/T		rs28580074
c.6903G→C	G2301G	29 G/C, 4 C/C		rs11740250
c.7636G→A	A2546T	11 G/A		[32, 52]

^1 ^References are given only for variants without a RefSNP accession ID (rs number)

### *NSD1 *microdeletion analyses

There was no evidence for partial or whole gene deletions or duplications of the *NSD1 *gene in any of the patients screened with MLPA.

## Discussion

Screening of the *NSD1 *gene in a cohort of 88 familial and sporadic cases of autism and macrocephaly did not reveal any intragenic mutations or deletions, indicating that *S*otos syndrome is a rare cause of autism spectrum disorders. The three missense variants identified in one individual each (R604L, S822C and E1499G), were inherited from unaffected parents, suggesting that they are likely to be polymorphisms. In addition, none of these variants is located in a known functional domain [28], further suggesting that they are nonpathogenic. Previous studies have shown that missense mutations are pathogenic only if they occur within functional domains of the protein involved in chromatin regulation [4].

Our negative findings are in agreement with recent studies showing that virtually all patients with a *NSD1 *mutation or deletion have the characteristic facial features of Sotos syndrome (broad forehead, sparse frontotemporal hair, long narrow face, malar flushing, down-slanted palpebral fissures, prominent jaw, pointed chin) [4,29,30]. However, the facial features are subtle and are difficult to recognize by clinicians with limited experience with this condition. The patients in our study presumably did not have the facial gestalt of Sotos syndrome, although most had not been evaluated by a clinical geneticist and it is likely that the psychiatrists that evaluated the patients were not all familiar with Sotos syndrome because of the relative rarity of this condition.

The other key clinical features of Sotos syndrome are the macrocephaly and the developmental delay. HC and height are increased in the majority of children with Sotos syndrome. By adulthood, height may fall within normal limits, but macrocephaly usually persists. However, recent studies have shown that HC and height are normal in 10% of *NSD1 *mutation-positive patients, indicating that overgrowth, previously considered as a major criterion of the disorder, is not necessary for the diagnosis of Sotos syndrome [4].

Our series included a high proportion of familial cases of autism spectrum disorders, as compared to singletons (51 vs. 37). Because Sotos syndrome is mostly a sporadic disorder, this could have contributed to the negative results of this study. However, several familial cases of Sotos syndrome with *NSD1 *mutations have been described in the past years [4,27,30-35], suggesting that familial, usually milder, forms of the disorder might be underdiagnosed.

Besides Sotos syndrome, there are several other syndromes presenting with macrocephaly and developmental delay, which are sometimes associated with autism. Among the better known is the fragile X syndrome, which is the most frequent genetic disorder identified in patients with autism spectrum disorders, accounting for about 2% of cases [36]. Molecular analysis for fragile X is routinely performed in patients with autism and mental retardation, and was ruled out in the patients participating in our study. Another disorder associated with autism and macrocephaly is the *PTEN *hamartoma tumor syndrome, which includes Cowden syndrome and Bannayan-Riley-Ruvalcaba syndrome. The *PTEN *gene has recently been found to be mutated in several children with autism spectrum disorders and macrocephaly [37-40]. We screened *PTEN *for mutations and deletions in the 88 individuals with autism spectrum disorders and macrocephaly reported in this study and identified one patient with extreme macrocephaly carrying a *de novo *missense mutation [25].

Other examples of disorders associated with macrocephaly which carry an increased risk for the development of autism include neurofibromatosis type I [41,42] and 22q13 deletions [43]. In addition, there are several reports in the literature of cases with progressive postnatal macrocephaly with autism, marked speech delay and mental retardation, referred to as the Cole-Hughes macrocephaly syndrome [44,45]. The Orstavik syndrome, described by Orstavik et al. [46], is characterized by macrocephaly, epilepsy, autism, mental retardation and dysmorphic features. Several other cases of the same syndrome were reported later by another group [47]. As Sotos syndrome, these disorders account for only a small number of patients with autism and macrocephaly.

The macrocephaly observed in about 20% of patients with autism appears to be an independent clinical trait, not related to sex, presence of morphological abnormalities, IQ, occurrence of seizures, or severity of autistic symptoms [20,21]. Converging evidence from HC measurements, MRI studies and postmortem brain weight indicates that an even greater proportion of children with autism have an abnormal regulation of brain growth, resulting in enlarged brains during early childhood [48]. At birth, HC is typically normal or slightly reduced, followed by accelerated growth during the first years of life. This early phase of excessive growth is followed by slowed growth after 2–4 years of age, so that in adolescents and adults HC measures are usually within normal range [48]. Other studies, however, have found increased brain volume in older populations of individuals with autism [49,50], so the timing of brain enlargement is not settled yet. Similarly, the pattern of enlargement across the brain lobes and cerebellum and the involvement of gray versus white matter remain unclear at present. Elucidation of the mechanisms involved in the pathological postnatal brain overgrowth may prove critical for understanding the emergence of autistic symptoms during the same time frame.

## Conclusion

In conclusion, no *NSD1 *mutations, 5q35 microdeletions or partial *NSD1 *deletions were identified in this large sample of patients with autism spectrum disorders and macrocephaly. Our results suggest that Sotos syndrome is a rare cause of autism spectrum disorders and that screening for *NSD1 *mutations and deletions in patients with autism and macrocephaly is not warranted in the absence of other features of Sotos syndrome.

## Competing interests

The author(s) declare that they have no competing interests.

## Authors' contributions

JDB and CB conceived the study, designed and coordinated it, performed part of the molecular genetic studies, and wrote the manuscript. GC and JG participated in the molecular genetic studies. GN, PC, RD, MR, JMS, EH, CG and ML participated in the recruitment of families. JMS, EH, CG and ML supervised the clinical evaluation of families. All authors read and approved the final manuscript.

## Pre-publication history

The pre-publication history for this paper can be accessed here:


